# Auditing the Audits: A Systematic Review on Different Procedures in Telemedicine

**DOI:** 10.3390/ijerph20054484

**Published:** 2023-03-02

**Authors:** Davide Cardile, Francesco Corallo, Irene Cappadona, Augusto Ielo, Placido Bramanti, Viviana Lo Buono, Rosella Ciurleo, Maria Cristina De Cola

**Affiliations:** IRCCS Centro Neurolesi Bonino-Pulejo, S.S. 113, Via Palermo C/da Casazza, 98124 Messina, Italy

**Keywords:** telemedicine, telehealth, telecare, audit, feedback, EASY-NET

## Abstract

Telemedicine is a process of delivering health care using information and communication technologies. Audit and feedback (A&F) constitute a systematic intervention that is aimed at collecting data, which are subsequently compared with reference standards and then returned to health care operators through feedback meetings. The aim of this review is to analyse different audit procedures on and by mean of telemedicine services and to identify a practice that is more effective than the others. Systematic searches were performed in three databases evaluating studies focusing on clinical audits performed on and by means of telemedicine systems. Twenty-five studies were included in the review. Most of them focused on telecounselling services with an audit and a maximum duration of one year. Recipients of the audit were telemedicine systems and service users (general practitioners, referring doctors, and patients). Data resulting from the audit were inherent to the telemedicine service. The overall data collected concerned the number of teleconsultations, service activity, reasons for referral, response times, follow-up, reasons why treatment was not completed, technical issues, and other information specific to each telemedicine service. Only two of the considered studies dealt with organizational aspects, and of these, only one analysed communicative aspects. The complexity and heterogeneity of the treatments and services provided meant that no index of uniformity could be identified. Certainly, some audits were performed in an overlapping manner in the different studies, and these show that although attention is often paid to workers’ opinions, needs, and issues, little interest was shown in communicative/organizational and team dynamics. Given the importance and influence that communication has in teamwork and care settings, an audit protocol that takes into account intra- and extra-team communication processes could be essential to improving the well-being of operators and the quality of the service provided.

## 1. Introduction

Telemedicine, Audit and Feedback

The term “Telemedicine” (TM) refers to a mode of delivering healthcare services through the use of innovative technologies. When two or more individuals are not in the same location or are constrained in their movements, they can make use of telemedicine. This can occur both between health professionals and between the health professional and the patient. Since the process may also involve at-home patient self-care and self-monitoring regimens, the term TM is used interchangeably with “telecare” and “telehealth” [1]. The first case of telemedicine utilization dates back to 1906 when Dutch physiologist W. Einthoven carried out trials of remote electrocardiographic consultation using a telephone [2]. Due to the inadequate communication systems of that period, the experiment proved ineffective [3], but today TM is widely used. The rapid progress that has been made in the information technologies field [4] has allowed this health service to complement and support traditional medicine in four different modalities:

1. Teleconsultation: This term can refer to two distinct processes.
  - Remote counselling between health professionals from different areas of intervention. Consultation often occurs because of the absence of a medical specialist and concerns a patient’s diagnosis or treatment [5].
  - Remote real-time medical examination in which one or more physicians assess a patient’s state of health using information technologies. Currently, there are two main options to deliver this kind of visit: purchasing a solution from a vendor and installing the study premises or buying video conferencing as a service from a vendor [6]. The first option allows easier management and better security control of devices, but the second option (the most popular one) involves:

2. Remote cooperation: In emergencies or critical scenarios, one physician helps and cooperates with another online through real-time video and audio communication. These scenarios could include both the recent COVID-19 pandemic [7] and operating room surgery [8,9]. In this latter case, the surgical team operates on the patient, while the remote participants can collaboratively work thanks to high-quality video-audio streams.
3. Telemonitoring: The periodic biomedical remote monitoring of the patients from a tablet, smartphone, or computer web browser is connected with low-cost and basic medical devices such as blood pressure cuffs, thermometers, and digital scales [10].

These modalities make telemedicine capable of creating new opportunities and improving the health service delivered, bringing several benefits. In remote areas poorly connected to major cities, TM can greatly increase the equity of access and the availability of skilled health care [11] and facilitate patients’ engagement in their own care [12,13]. Furthermore, telemonitoring can improve the quality of life in chronic patients through self-management solutions and improving self-care, patient education, treatment adherence, and survival [14]. Several studies suggest that TM seems to be cost-effective in that it reduces hospital admissions and improves the management of chronic disease and patient compliance [15].

Despite all these great benefits, TM is not without problems and limitations. Indeed, medical and health service researchers have begun to note that telemedicine initiatives frequently fail to achieve uptake or to demonstrate significant benefits to patients [16,17,18]. Factors such as the lack of integration and interoperability of computing systems and software, a failure to achieve cost-effectiveness, and the negative attitudes to telemedicine on the part of medical practitioners have been identified as barriers to successful telemedicine initiatives [16,18,19].

These can be traced back to both technical and human factors. To evaluate an entire TM process (even from different perspectives) and highlight any critical issues, an audit can be used.

An audit is a process of systematic data collection and comparison that seeks to improve the quality and outcome of different processes or practices through the modification of dysfunctional parameters [20].

This term is often used accompanied by attributes that indicate specific approaches to assess the quality of care or other aspects of the evaluated process:

- Economic-managerial audit: aims to assess the appropriateness and efficiency of planning, the management of resources, and expenditure.
- Organizational audit: evaluates organizational processes (e.g., information system, workload assessment, organisational procedures aimed at the acquisition and support of technologies). System audit: examines the main organisational aspects, practices, procedures, and controls that support the efficiency of an entire organisation, a hospital, or a department. Clinical audit: is an intervention aimed at collecting and comparing clinical data with reference standards [21,22]. It is fundamental to improving clinical practice and equity in different settings.

Beyond these approaches, an audit can be internal or external. In the former case, an organisation (e.g., a hospital) is reviewed by an internal group of experts who evaluate its performance to identify possible improvements. On the other hand, an external audit is performed by professionals who are not associated with the assessed service, facility, or treatment.

An audit multidisciplinary team provided a series of quality indicators on which data are collected and then compared with the reference standards. Subsequently, any resulting deviation from these standards is communicated to professionals in a structured phase called “feedback”. This causes the audit to be conceived as a quality improvement process that aims to enhance patient care and outcomes.

The aim of this systematic review is to analyse different audit procedures on and through the means of telemedicine services. 

## 2. Materials and Methods

A systematic review of currently published studies was performed following the standard guidelines. Online database searches were performed for articles published before 7 October 2022 on audits performed on and by telemedicine systems. The literature search was conducted via PubMed, Cochrane Central Register of Controlled trials, and Web of Sciences. It was carried out using the following search keywords and terms: (‘telemedicine’ OR ‘telehealth’ OR ‘telecare’ AND ‘audit’).

Inclusion criteria

A study was included if it investigated a clinical audit performed on or through the means of telemedicine systems. Only articles written in English were included in the review. 

Exclusion criteria

A study was excluded if there was a lack of telemedicine systems used and if it was a dissertation, commentary, letter, or editorial. Systematic, integrative, or narrative reviews were also excluded, although their reference lists were checked and included if appropriate. No restriction due to the year of publication was adopted since the development of telemedicine is recent.

## 3. Results

**Figure 1:**
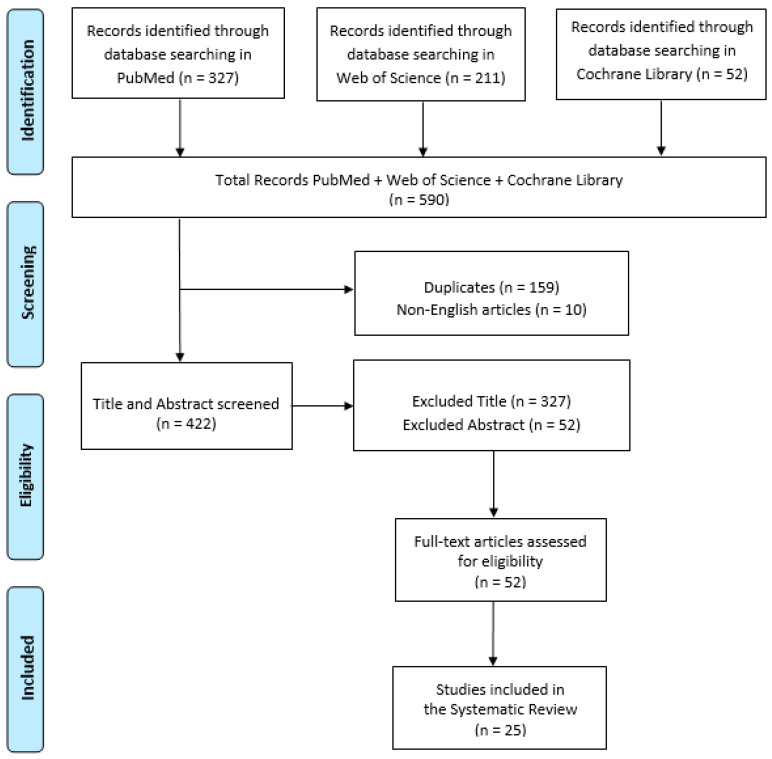
PRISMA flow chart for the current systematic review.

The initial electronic data search yielded a total of 590 potentially relevant studies (327 results on PubMed, 211 on Web of Science, and 52 on Cochrane Library); of these, 159 were duplicates, and 10 were non-English language articles (Figure 1). 

Of the resulting 422 articles, 327 were excluded by title and 52 by abstract. The total of full-text articles that were assessed for eligibility was 51, and the number of studies included was finally 25. Of this sample of the studies considered, eleven were retrospective, three prospective, and eleven were unspecified (Table 1 and Table 2). All articles included different clinical diseases and medical disciplines: dermatology [23,24,25,26], sexual health [27], psychiatry [28], neurology [29,30], geriatrics [31], ophthalmology [32,33,34,35], physiology [36], orthopaedics [37,38,39], respiratory failure [40], paediatric diabetic [41], injury/illness [42], mixed [43,44], radiology [45], and atrial fibrillation [46], N.S. [47].

### 3.1. Telemedicine Service

Services treated by studies were different: 16 were telecounselling, four were telemonitoring [40,44,45,46], and five were mixed [26,31,37,38,41]: telecounselling + telemonitoring.

The electronic information and/or telecommunication technologies employed included video call/conference (*n* = 6), email (*n* = 2), instant messaging (*n* = 1), network (*n* = 7), Telephone calls (*n* = 3), Image posting (*n* = 1), Electronic clinical assessment form (*n* = 1), app (*n* = 2), mixed (*n* = 3).

### 3.2. Studies Data

Recipients of the audit performed by each study taken into account included telemedicine systems and service users. The latter category included general practitioners (GP), referring doctors, and patients.

The duration of the studies analysed by this review ranged from one month to eight years, but most of them (*n* = 14, 56%) took place within a maximum period of one year. Of course, all the studies focused their attention on data inherent in the telemedicine service. The overall data collected concerned: the number of teleconsultations, service activity, the reason for referral, response times, follow-up, reasons why treatment was not completed, technical issues, and other information specific to each telemedicine service.

### 3.3. Satisfaction

In five out of the twenty-five studies [23,24,41,42,44], the satisfaction of users taking part in services was assessed (Table 3 and Table 4). They could be both patients and physicians. Almost all respondents were satisfied or highly satisfied with the service; most of them found it useful and said they would use it again. They found TM to be a process that:

– Makes it easier to start treatment for the patient [32,41]
– A good way to provide care for young people in rural sites [23,32,41]
– Very helpful, especially in the peripheries, to cut on travelling and waiting time, thus saving money [41].
– Enabled ready access for specialist services [23,41,42]
– Empowered local clinics, reducing the load on the hospital health care professionals [41,44].

### 3.4. Feedback

In one of the articles mentioned above [44], an A&F process was given via telemedicine. Specifically, in this article, participants received continuous feedback via a specific application. These healthcare professionals found this kind of feedback a valuable way to improve clinical practice; in fact, according to their opinion:

– It increases the skills of healthcare workers through the proper communication of errors made.
– It increases awareness of the implications of making mistakes.
– It allows the development of critical thinking and improved decision-making process.
– In addition, it has emerged that it is not only important to communicate the error but also the timing and manner in which this occurs.

### 3.5. Organizational Aspects

The organizational aspects of telemedicine systems were evaluated in only two studies (8%). One of these two articles [40] took into account only the time of staff activity, while the other [31] specifically evaluated differences in the workflow of operators, training, changes in culture, attitude, and management. The latter was the only study in the sample to consider communicative aspects in the members of the healthcare team.

Data pertaining to the economic part were collected in 5 studies [26,31,39,40,46]. One of them included economic aspects as one of the indicators to be evaluated, but then no data were recorded because it was not in line with the aims of the study [31]. Another article [46] evaluated cost-effectiveness, but in reference to drug treatment. Using the telemedicine service shows a reduction in costs by 23% [39] and 39% [40]. Furthermore, avoided transfer and hospitalizations resulted in an overall total savings of AUD 1,892,584, or AUD 6460 per patient [26].

Within the sample, four studies were considered [28,34,36,38] as conducted during the COVID-19 pandemic. Of these two [28,38] was a confrontation between the TM service before and during the COVID-19 pandemic.

### 3.6. Models

Some of the studies analysed in this paper have referred to specific practical-theoretical models that are aimed at bringing about concrete and measurable change.

Owen et al. [36], in their study, adopted the RE-AIM framework: a planning and evaluation model that addresses five dimensions of individual and setting-level outcomes that are important to program impact and sustainability [48]. The above five dimensions are: reach the target population, effectiveness, adoption by target staff, settings, systems and communities, implementation costs and adaption, maintenance/sustainment of intervention effects in individuals, and settings over time (https://re-aim.org/learn/what-is-re-aim/, accessed on 22 November 2022).

Haydon et al. [31] used the model for assessment of telemedicine (MAST): an evaluation framework that focuses on the measurement of effectiveness and quality of care [49]. The MAST represents a multidisciplinary process, evaluating the medical, social, economic, and ethical aspects of telemedicine in a systematic, unbiased, robust manner [50].

The use of MAST includes three steps [51]. In the “preceding assessment” (Step 1), the maturity of TM technology and the organization using the device is assessed. If the maturity of the technology/organization using the TM service needs to be developed further, formative studies, including participatory design (PD)/usability/feasibility studies, must be carried out. Following, a “multidisciplinary assessment” (Step 2) could be carried out to analyse seven domains, including the identification of health problems and the characteristics of the application; safety; clinical effectiveness; patient perspectives; economic aspects; organisational aspects; and socio-cultural, ethical, and legal aspects. Finally, an assessment should be made of the transferability of the results (Step 3) reported in studies concerning the previous steps.

## 4. Discussion

The aim of this review was to identify an audit procedure that was more widely used or more effective than others. However, the complexity and heterogeneity of the treatments and services provided and examined by each study did not allow for the identification of a uniformity index. Certainly, some audits are performed in an overlapping manner in the different studies, and these show that although attention is often paid to workers’ opinions, needs, and issues, little interest was shown in communicative/organizational and team dynamics. Kapoor et al. [52] found that human factors are most responsible for the successful outcome of their telemedicine project. In fact, supporting a telemedicine program can require a unique set of skills compared to a standard consultation. Several studies also elicit the importance of communication within teamwork, especially when this occurs within a healthcare setting [53,54,55]. There is a need for someone who can interface with the clinical staff without being out of their depth: this person will obviously need to be an excellent communicator and should have the ability to speak in a plain, nontechnical language that end users can understand [6]. Communication and interpersonal skills turn out to be crucial, especially in highly time-dependent contexts such as emergency-urgency departments [56].

Furthermore, in a study that analysed TM from an organizational perspective [57], authors found that a dearth of perceived central leadership, a lack of information about the technology itself, poorly designed and cumbersome means for scheduling and utilizing telemedicine technology, the absence of explicit strategic goals, and poor communication in introducing this technology to medical personnel and to the public would undermine the telemedicine process.

Then, it might be useful and interesting to identify a standardized audit procedure that takes into account some cross-disciplinary and psychological aspects, such as communication. In this regard, Hargie and Tourish [58] in 1993 proposed an audit methodology for communication. First, since performing this process on a very large organization or telemedicine service can raise ethical and economic difficulties that may even interfere with the daily functioning of most organizations, they suggest a full and comprehensive audit, but for a small section of an organization or of a TM service. In general, they refer to ‘the use of certain survey instruments’. For example, they suggest validated questionnaires and/or semi-structured interviews to evaluate current communication practices, identify areas of strengths and weakness, and suggest how improvements could be affected [59,60]. Subsequently, audio or video recordings of interpersonal encounters could be analysed and evaluated, as well as live observations of such episodes in situ. Another simple and readily available instrument is diary analysis, through which it is possible to obtain information concerning communication contacts over a set period of time.

## 5. Conclusions

Audit and feedback constitute a fundamental process for controlling and improving the quality of service. Data collection and comparison with reference standards take place in depth, especially with regard to the treatment process and the results obtained. However, elements that have a strong impact on the process and, consequently, on its results are often overlooked. These elements include communication, motivation, organisation, work-related stress, and operators’ coping skills. This is why it would be useful to broaden the concept of “quality” by delving more deeply into the psychological aspects related to the staff and their work.

Future studies might include a wider range of keywords in order to explore further aspects of this challenging topic. Moreover, an effective and universally recognized set of targeted audit protocols could be developed based on future research focused on refining, updating, and standardizing existing procedures to evaluate organizational aspects such as communication.

## Figures and Tables

**Table 1:** Description of studies performed on a telemedicine service.

Study	Type	Aim	Disease	Telemedicine System	Population	Duration
Morris C., Scott E., Mars M., 2022 [23]	Retrospective	Auditing the informal use of instant messaging for dermatology by: Assessing the nature and content of the information sent to dermatologists. Determining the referring doctors’ interactions and satisfaction with their use of instant messaging.	Dermatology	Telecounselling	Service Users Medical Specialist (Dermatologists) Referring doctors	4 years and 3 months
Biscak T.M. et al., 2013 [24]	Retrospective	To assess the use of the teledermatology service, including the characteristics of clinicians using the service and their perceptions of it.	Dermatology	Telecounselling	Consultations Referring doctors	1 year
Miller B.J. et al., 2020 [25]	Retrospective	Evaluate a telemedicine system that provides a real-time videoconference teledermatology clinic to enable patients in rural and remote Queensland to access a specialist for dermatology care.	Dermatology	Telecounselling	Consultations	2 years
McWilliams T. et al., 2016 [26].	Retrospective	To conduct a retrospective audit of avoided transfers and bed days as a result of the use of the paediatric Burns Telehealth Service and to estimate their cost savings in 2012/13.	Pediatric burn	Telecounselling Monitoring	Caregivers of patients	8 years
Biggs K. et al., 2010 [27]	Retrospective	Evaluate an “Email a Clinician” link on a medically reviewed sexual health website which allows general practitioners to communicate remotely with sexual health clinic specialists.	Sexual Health	Telecounselling	General Pratictioners Health care Workers	1 year
NG L., Narayanan N., Diamond D., Pitigala N., 2022 [28]	N.A.	To identify service user demographic and clinical characteristics of an acute mental health service in South Auckland during the first New Zealand coronavirus.	Psychiatry	Telecounselling	Service Users	1 Month (comparison)
Harvey R. et al., 1998 [29]	Retrospective	To audit and evaluate the introduction of a novel support service for younger people with dementia, their families, and the professionals caring for them.	Neurology	Telecounselling	Service Users	2 year
Handschu et al., 2014 [30]	N.A.	The implementation and certification of a single quality management system for stroke care in all participating hospitals of the network, including stroke centers and local hospitals.	Neurology (Stroke)	Telecounselling	Consulting PhysiciansPhysicians in local hospitals Patients	4 years
Haydon H.M. et al. 2021 [31]	N.A.	Investigate the growth and reach of Geri-Connect: a service established in 2017 to support people living in residential aged care facilities in regional Victoria, Australia.	Geriatry	Telecounselling Telemonitoring	Clinicians	3 years
Kennedy C. et al., 2006 [32]	N.A.	To audit a web-based telemedicine service in ophthalmology.	Ophthalmology	Telecounselling	Service Users	1 year
Patel A. et al., 2020 [33]	N.A.	Assess the efficacy of the telephone service offered by the Moorfields Eye Hospital NHS Foundation Trust (London) during the COVID-19 pandemic.	Ophthalmology	Telecounselling	Consultations Service Users	//
O’Day et al., 2016 [34]	Prospectivee	Comparing two five-month prospective audits of a teleophthalmology service (Lions Outback Vision) in rural Western Australia.	Ophthalmology	Telecounselling	Consultations	2 years (comparison)
Bartnik et al., 2018 [35]	Retrospective	Audit the lion’s outback vision and provide practical insights for others looking to embed a telemedicine program as part of delivering outreach clinical services.	Ophthalmology	Telecounselling	Consultations	1 year
Owen J. et al., 2022 [36]	Retrospective	To examine the impact of the COVID-19 pandemic on the reach, efficacy, adoption, and implementation of telehealth delivery for the exercise of physiology services.	Physiology	Telecounselling	Physiology clinicians	5 months
Navein J., Hagmann J., Ellis J. 1997 [37]	Retrospective	Evaluate a satellite-based telemedicine system deployed in support of remote primary-care physicians in the U.S. military.	Orthopedics	Telecounselling Monitoring	Referring/Consulting clinicians Patients	1 year and 3 months
Dunkerley S. et al., 2020 [38]	Prospective	Assess how the initial changes implemented within the department had affected outpatient fracture management and evaluate if the structure was meeting the new standards set by BOAST (the British Orthopedic Association).	Fracture	Telecounselling Monitoring	Referring/Consulting clinicians Patients	1 month
Beard M., Orlando J.F., Kumar S. 2017 [39]	Prospective	Determine the feasibility, appropriateness, and access to a telehealth clinic by auditing the process and outcomes by a pilot telehealth spinal assessment clinic.	Spinal disorders	Telecounselling	Consultations	5 months
Vitacca et al., 2010 [40]	Retrospective	Evaluate patients’ characterization and staff workload on a teleassistance service for patients with chronic respiratory failure.	Respiratory failure	Monitoring	Patients	5 years
Williams M. 2020 [41]	Retrospective	To evaluate rural paediatric diabetic telehealth clinics, including whether they meet clinical standards and provide equivalent care to central clinics: families were satisfied, and difficulties were encountered.	Pediatric diabetic	Telecounselling Monitoring	Referring/Consulting clinicians Patients	1 year
Webster et al., 2008 [42]	N.A.	Evaluate a telemedicine service established for the Scottish Police College with medical advice provided from the Aberdeen Royal Infirmary,	Injury/illness	Telecounselling	Consultations Service Users	1 year and 4 months
Alkmim M.B.M et al., 2015 [43]	N.A.	To describe the audit of the teleconsultation responses performed by the Telehealth Network of Minas Gervais (TNMG).	Mixed	Telecounselling	Service Users	1 month
Jury S.C., Kornberg J.A. 2016 [47]	N.A.	Evaluate the possibility of integrating telehealth into “business as usual”.	N.S.	Telecounselling	Clinicians	1 month

**Table 2:** Description of studies where audit or feedback is provided via a telemedicine service.

Study	Type	Aim	Disease	Telemedicine System	Population	Duration
Keyworth C. et al., 2017 [44]	N.S.	Evaluate the acceptability and feasibility of providing feedback via MyPrescribe: a mobile-compatible website. Analyze and discuss the findings in the context of the COM-B model. Outline a series of practical implications and recommendations for using MyPrescribe.	Mixed (heart care, renal transplant/renal, gastroenterology	Tele monitoring	Pharmacists Junior doctors	4 months
Morozov S. et al., 2018 [45]	N.S.	Evaluation of a telemedicine-based peer review of computed tomography and magnetic resonance imaging to enhance quality management in radiology.	Radiology Mixed	Tele monitoring	Patients	12 Months
Orchard J. et al., 2020 [46]	N.S.	Cost-effective Analysis of eHealth Tools to Support All Stages of Screening.	Atrial fibrillation	Tele monitoring	Patients	11 months

**Table 3:** Description of the audit features included within the studies performed on telemedicine services.

Study	Procedure	Recipients	N	Collected Data
Morris C., Scott E., Mars M., 2022 [23]	An audit was performed to assess the nature and content of the information sent to dermatologists by referring doctors. A 43 item questionnaire was administered to collect information related to the service.	Service Users Medical Specialist (Dermatologists) Referring doctors	1034 messages sent. 830 answers received. 81 responses to questionnaires	Demographics, technical issues response times, satisfaction, consultations, consent and guidelines, data security.
Biscak T.M. et al., 2013 [24]	An audit was conducted to evaluate all teledermatology consultations. A retrospective questionnaire was administered to obtain feedback from the clinicians who used the service during the audit period.	Consultations Referring doctors	685 emails analysed 34 responses to questionnaire	Consultation diagnosis satisfaction
Miller B.J. et al., 2020 [25]	An audit of the tele dermatology clinic was performed via a retrospective chart review of all referrals to the clinic for a two-year period (September 2015 to October 2017).	Consultations	483 consultations for 178 patients.	Demographics, service activity and wait times, urgency category assigned to videoconferencing patients, diagnoses
McWilliams T. et al., 2016 [26]	A retrospective chart audit identified activity, avoided unnecessary acute and scar review patient transfers, inpatient bed days, and their associated avoided costs to the tertiary burn unit and patient travel funding.	Caregivers of patients	904 patients	Avoided unnecessary acute and scar review, patient transfers, inpatient bed days and their associated avoided costs to the tertiary burn unit.
Biggs K. et al., 2010 [27]	Every email sent to the web-linked email account was identified and analyzed to determinate its content and sender classification.	General Practitioners Health care workers	324 emails analysed.	Email nature and information requested by the sender
NG L., Narayanan N.,Diamond D., Pitigala N., 2022 [28]	A clinical audit was conducted on data from electronic psychiatric clinical assessment forms, to compare two randomly selected group (2020 vs. 2019 group)	Service Users	413 service users during 2020. 785 service users during 2019.	First group: Demographics, Technology Diagnoses, psychological issues, follow-up. Second group: Same 1st group variables plus psychological COVID-19 related issues.
Harvey R. et al., 1998 [29]	General information was collected on every caller. Calls were divided into two main groups: a “registered” group and a “generic” one. Only the first group received specific clinical advice via a letter to their GP.	Service Users	1121 calls received	Demographics, relationship between caller and patient, consultation
Handschu et al., 2014 [30]	Data recording by teleconsultations and all thrombolysis cases, as well as technical data and service complaints within the network, was implemented.	Consulting Physicians Physicians in local hospitals Patients	2049 stroke teleconsultation in 2009 2324 patients in 2011 4517 patients seen in all local hospital.	Demographics, Consultation, Thrombolysis procedure informations. Deviations/key notes for improvement in internal audits from 2009 to 2011.
Haydon H.M. et al., 2021 [31]	Geri-Connect service activity data (2017–2020) were analysed to investigate the growth and reach of the service. The Model for the Assessment of Telemedicine (MAST, with these domains: Health problem and characteristics, clinical effectiveness, economic, organisational, scalability and generalisability). Semi-structured interviews with key stakeholders provided staff perspectives on the utility and barriers.	Clinicians	Data resulting for each of the 5 MAST domain. 10 phone interviews	Health problem and characteristic, clinical effectiveness, staff perception of patients, organizational aspects, scalability, generalizability.
Kennedy C. et al., 2006 [32]	Telemedicine website activity was monitored by a site supervisor. On this site referring doctors could upload brief case histories, problems, and treatment to seek the advice of a specialist.	Service Users	132 cases posted to the website	Number, types of cases and additional information were analysed.
Patel A. et al., 2020 [33]	An audit of 500 consecutive telephone low vision appointments was performed. The successful completion of the assessment and clinical outcomes (low vision aids prescribed, onward referral) were recorded.	Consultations Service Users	364 completed telephone low vision assessment	Participants diagnosis, Consultations. Patient lifestyle information. Social situation, employment and education.
O’Day et al., 2016 [34]	Two five-month audits were conducted. The first was conducted prior to the implementation of the intervention (2012), while the second was conducted during the period of intervention (2014).	Consultations	Number of video-consultations performed between the 2 audit periods	Differences between the 2 audits in: demographics, diagnosis, consultation, follow-up plan and technology
Bartnik et al., 2018 [35]	A retrospective audit was performed through the use of a data extraction tool to record information from all tele ophthalmology consultations performed in the time period (2015–2016).	Consultations	709 patients referring to the service. 683 teleconsultations.	Diagnosis, consultations, cataract surgery rate, remoteness area of patients referred and imaging accompanying the referral.
Owen J. et al., 2022 [36]	A retrospective audit of exercise physiology services was conducted comparing Australian practises before (prior to 25 January 2020) and during the COVID-19 pandemic (after 25 January 2020). Relevant dimensions of the RE-AIM (reach, effectiveness, adoption and implementation) framework were adopted.	Physiology clinicians	80 online survey completed	Clinicians and patients demographics, usual practise (prior to 25 January 2020) and changes to practise (after 25 January 2020), Consultations, Technology.
Navein J., Hagmann J., Ellis J. 1997 [37]	Data were collected using evaluation forms that were completed by referring and consulting physicians at the time of the consult and through structured interviews with referring physicians.	Referring/Consulting clinicians Patients	53 consults 47 patients by seven GMOs/PAs	Demographics, technology and questions about: change in diagnosis/treatment and change in evacuation status resulting from the consult, improvement in the referring physician’s confidence, improvement in the military effectiveness of the unit.
Dunkerley S. et al., 2020 [38]	After the individuation of the four most relevant BOAST standards: an audit was designed around fracture immobilisation, the type of initial fracture clinic assessment, the default virtual follow-up clinic, and late imaging. Interventions were implemented and re-audited.	Referring/Consulting clinicians Patients	223 patients	Emprovement in BOAST standards
Beard M., Orlando J.F., Kumar S. 2017 [39]	Data were recorded from all consultations managed using a videoconferencing technology between the Royal Adelaide Hospital and Port Augusta Community Health Service, South Australia between September 2013 and January 2014. Data were compared to a previous SAC clinic between August and December 2012.	Consultations	41 participants in the SAC-T and 22 consultations in the SAC-O.	Analysis of process, service activity, clinical actions, safety and costs, demographics, clinical details.
Vitacca et al., 2010 [40]	Administrative and medical records from the TA service were retrospectively reviewed. Patients’ records were taken from the electronic database of the ‘‘Salvatore Maugeri Foundation’’ TA Service in Lumezzane (Brescia). Administrative and medical records from the TA service were retrospectively reviewed.	Patients	396 consecutive CRF patients were analyzed.	Demographics, call characteristics, time of staff activity, salary cost evaluation, cost of the service.
Williams M. 2020 [41]	An audit of a telehealth service for children and adolescents with type 1 diabetes mellitus at four rural sites. A feedback survey was administered to satisfaction with the telehealth service, with preference for in-person or telehealth consultation, and any concerns regarding deficits in care and suggestions for improvements.	Referring/Consulting clinicians Patients	19 children and adolescents aged 2–17 years were seen in the rural telehealth clinics.	Growth, information, and requirements of telehealth. Satisfaction, preference for in-person or telehealth consultation, deficit in care and suggestions for improvements.
Webster et al., 2008 [42]	Data collection forms were constructed and used at the Police College and in Aberdeen from November 2004 to February 2006. Problems were classified as either illness or injury, based on the main presenting complaint. In addition, the Police College conducted an informal interview with people who had used the service.	Consultations Service Users	192 patients, 97 teleconsultations. 66 satisfaction interviews for students and staff	Date of presentation, date of injury, problem type, management, treatment, follow-up and referral for physiotherapy. Satisfaction.
Alkmim M.B.M et al., 2015 [43]	A random sample was selected from medical and non-medical teleconsultations performed by the specialists from the TNMG. All responses were scored according to their impact on the quality of the teleconsultation.	Service Users	640 teleconsultation responses (medical:76% non-medical: 24%)	Objectivity, quality, ethics, courtesy
Jury S.C., Kornberg J.A. 2016 [47]	A one-month audit of booked telehealth was completed, looking for anything that impacted on the delivery or billing of telehealth. Each clinician was also asked for feedback on the day of their consultation.	Clinicians	125 clinician feedback	Clinician feedback on telehealth appointments, Billing, Scheduling, and requesting telehealth.

**Table 4:** Description of audit features included in studies where the audit or feedback was performed via a telemedicine service.

Study	Procedure	Recipients	N	Collected Data
Keyworth C. et al., 2017 [44]	Pharmacists were invited to collect prescribing error data for junior doctors. They were invited to take part in semi-structured interviews exploring the perceptions of the acceptability and feasibility of MyPrescribe as a training tool aimed at improving prescribing practices.	Pharmacists (*n* = 11) junior doctors (*n* = 52)	200 prescribing errors Interviews	Demographics, medical specialty. Change in COM-B model (capability, opportunity, motivation, behavior). Opinions on telemedicine service.
Morozov S. et al., 2018 [45]	A group of experts, two or three for each record, performed a distant peer review. If one of the experts considered that the discrepancy was significant, the system sent the study to another expert. If even the second disagrees, the study is redirected for the final evaluation of the third expert.	Patients	23.199 studies	Quality control focuses on: Technical performance and detection of pathology. Scoring of the degree of discrepancy between clinical opinion
Orchard J. et al., 2020 [46]	GPs and/or practice nurses offered screening for AF with smartphone handheld single-lead ECGs (iECGs) (KardiaMobile) to eligible patients. To support the screening eHealth tools information was extracted from patients’ electronic medical records and guideline recommendations were made regarding treatment.	Patients	3103 patients screened	Demographics, iECG screening, medication, and diagnostic information from the practices’ electronic patient records, treatment, cost- effectiveness.

## References

[1] Wilson L.S., Maeder A.J. (2015). Recent Directions in Telemedicine: Review of Trends in Research and Practice. Healthc. Inform. Res..

[2] Nesbitt T.S. (2012). The Evolution of Telehealth: Where Have We Been and Where Are We Going.

[3] Blackburn H. (1957). Translation of The Telecardiogram, article by W. Einthoven. Am. Heart J..

[4] Zanaboni P., Wootton R. (2012). Adoption of telemedicine: From pilot stage to routine delivery. BMC Med. Inform. Decis. Mak..

[5] Lai F. (2008). Robotic telepresence for collaborative clinical outreach. Stud. Health Technol. Inform..

[6] Baker J., Stanley A. (2018). Telemedicine Technology: A Review of Services, Equipment, and Other Aspects. Curr. Allergy Asthma Rep..

[7] Witowska-Zimny M., Nieradko-Iwanicka B. (2022). Telemedicine in Emergency Medicine in the COVID-19 Pandemic-Experiences and Prospects—A Narrative Review. Int. J. Environ. Res. Public Health.

[8] Bao X., Guo S., Xiao N., Li Y., Yang C., Shen R., Cui J., Jiang Y., Liu X., Liu K. (2018). Operation evaluation in-human of a novel remote-controlled vascular interventional robot. Biomed. Microdevices.

[9] Marescaux J., Leroy J., Rubino F., Smith M., Vix M., Simone M., Mutter D. (2002). Transcontinental Robot-Assisted Remote Telesurgery: Feasibility and Potential Applications. Ann. Surg..

[10] Barrett M., Combs V., Su J.G., Henderson K., Tuffli M. (2018). AIR Louisville: Addressing asthma with technology, crowdsourcing, cross-sector collaboration, and policy. Health Aff. (Millwood).

[11] Finkelstein S.M., Speedie S.M., Potthoff S. (2006). Home Telehealth Improves Clinical Outcomes at Lower Cost for Home Healthcare. Telemed. e-Health.

[12] Coulter A. (2012). Patient Engagement—What Works?. J. Ambul. Care Manag..

[13] Weinstein R.S., Lopez A.M., Joseph B.A., Erps K.A., Holcomb M., Barker G.P., Krupinski E.A. (2013). Telemedicine, Telehealth, and Mobile Health Applications That Work: Opportunities and Barriers. Am. J. Med..

[14] Planinc I., Milicic D., Cikes M. (2020). Telemonitoring in Heart Failure Management. Card. Fail. Rev..

[15] Rojas S.V., Gagnon M.P. (2008). A systematic review of the key indicators for assessing telehomecare cost-effectiveness. Telemed. J. E Health.

[16] Armfield N.R., Edirippulige S.K., Bradford N., Smith A. (2014). Telemedicine—Is the cart being put before the horse?. Med. J. Aust..

[17] Elwyn G., Hardisty A.R., Peirce S.C., May C., Evans R., Robinson D.K.R., Bolton C.E., Yousef Z., Conley E.C., Rana O.F. (2011). Detecting deterioration in patients with chronic disease using telemonitoring: Navigating the ‘trough of disillusionment’. J. Eval. Clin. Pract..

[18] Wootton R., Geissbuhler A., Jethwani K., Kovarik C., Person D.A., Vladzymyrskyy A., Zanaboni P., Zolfo M. (2012). Long-running telemedicine networks delivering humanitarian services: Experience, performance and scientific output. Bull. World Health Organ..

[19] May C.R., Finch T.L., Cornford J., Exley C., Gately C., Kirk S., Jenkings K.N., Osbourne J., Robinson A.L., Rogers A. (2011). Integrating telecare for chronic disease management in the community: What needs to be done?. BMC Health Serv. Res..

[20] Francesconi P., Bellini B., Furlan F. (2021). Audit & Feedback: Un esempio di utilizzo per migliorare l’aderenza alle terapie. Recent. Prog. Med..

[21] Cardile D., Ielo A., Corallo F., Cappadona I., D’Aleo G., De Cola M.C., Bramanti P., Ciurleo R. (2023). Communication Training: Significance and Effects of a Preliminary Psychological Intervention upon an Audit Team. Int. J. Environ. Res. Public Health.

[22] Ciurleo R., De Cola M.C., Agabiti N., Di Martino M., Bramanti P., Corallo F. (2022). Audit and feedback in cardio- and cerebrovascular setting: Toward a path of high reliability in Italian healthcare. Front. Public Health.

[23] Morris C., Scott R.E., Mars M. (2022). An Audit and Survey of Informal Use of Instant Messaging for Dermatology in District Hospitals in KwaZulu-Natal, South Africa. Int. J. Environ. Res. Public Health.

[24] Biscak T.M., Manoharan S., Eley R., Sinnott M., Soyer H.P. (2013). Audit of a State-wide store and forward teledermatology service in Australia. J. Telemed. Telecare.

[25] Miller B.J., Finnane A., Vun Y., Halloran S., Stapelberg A., Soyer H.P., Caffery L. (2020). Real-time teledermatology clinics in a tertiary public hospital: A clinical audit. Australas. J. Dermatol..

[26] McWilliams T., Hendricks J., Twigg D., Wood F., Giles M. (2016). Telehealth for paediatric burn patients in rural areas: A retrospective audit of activity and cost savings. Burns.

[27] Biggs K., Lowe P., Walsh J., Lagios K. (2010). Audit of a sexual health website email link for general practitioners. Int. J. STD AIDS.

[28] Ng L., Narayanan N., Diamond D., Pitigala N. (2021). Audit of acute psychiatric presentations during New Zealand’s first COVID-19 national lockdown. Australas. Psychiatry.

[29] Harvey R., Roques P.K., Fox N.C., Rossor M.N. (1998). CANDID--Counselling and Diagnosis in Dementia: A national telemedicine service supporting the care of younger patients with dementia. Int. J. Geriatr. Psychiatry.

[30] Handschu R., Scibor M., Wacker A., Stark D.R., Köhrmann M., Erbguth F., Oschmann P., Schwab S., Marquardt L. (2014). Feasibility of Certified Quality Management in a Comprehensive Stroke Care Network Using Telemedicine: STENO Project. Int. J. Stroke.

[31] Haydon H.M., Caffery L.J., Snoswell C.L., E Thomas E., Taylor M., Budge M., Probert J., Smith A.C. (2021). Optimising specialist geriatric medicine services by telehealth. J. Telemed. Telecare.

[32] Kennedy C., Bowman R., Fariza N., Ackuaku E., Ntim-Amponsah C., Murdoch I. (2006). Audit of Web-based telemedicine in ophthalmology. J. Telemed. Telecare.

[33] Patel A., Fothergill A.S., Barnard K.E.C., Dunbar H., Crossland M.D. (2021). Lockdown low vision assessment: An audit of 500 telephone-based modified low vision consultations. Ophthalmic Physiol. Opt..

[34] O’Day R., Smith C., Muir J., Turner A. (2016). Optometric use of a teleophthalmology service in rural Western Australia: Comparison of two prospective audits. Clin. Exp. Optom..

[35] Bartnik S.E., Copeland S.P., Aicken A.J., Turner A.W. (2018). Optometry-facilitated teleophthalmology: An audit of the first year in Western Australia. Clin. Exp. Optom..

[36] Owen P.J., Keating S.E., Askew C.D., Clanchy K.M., Jansons P., Maddison R., Maiorana A., McVicar J., Robinson S., Mundell N.L. (2022). Impact of the COVID-19 Pandemic on Exercise Physiology Services in Australia: A Retrospective Audit. Sports Med. Open.

[37] Navein J., Hagmann J., Ellis J. (1997). Telemedicine in Support of Peacekeeping Operations Overseas: An Audit. Telemed. J..

[38] Dunkerley S., Kurar L., Butler K., James M., Lowdon I. (2020). The success of virtual clinics during COVID-19: A closed loop audit of the British orthopaedic association (BOAST) guidelines of outpatient orthopaedic fracture management. Injury.

[39] Beard M., Orlando J., Kumar S. (2016). Overcoming the tyranny of distance: An audit of process and outcomes from a pilot telehealth spinal assessment clinic. J. Telemed. Telecare.

[40] Vitacca M., Bazza A., Bianchi L., Gilè S., Assoni G., Porta R., Bertella E., Fiorenza D., Barbano L., Comini L. (2010). Tele-assistance in chronic respiratory failure: Patients’ characterization and staff workload of 5-year activity. Telemed. J. e-Health Off. J. Am. Telemed. Assoc..

[41] Williams M. (2020). Using telehealth for rural paediatric diabetics: Does it deliver good care?. J. Paediatr. Child Health.

[42] Webster K., Fraser S., Mair F., Ferguson J. (2008). Provision of telehealth to the Scottish Police College. J. Telemed. Telecare.

[43] Alkmim M.B.M., Marcolino M.S., Maia J.X., Pessoa C.G., Machado E., Sousa L. (2015). Clinical Quality Control of a Large-Scale Teleconsultation Service. Stud. Health Technol. Inform..

[44] Keyworth C., Hart J., Thoong H., Ferguson J., Tully M., Murray J. (2017). A Technological Innovation to Reduce Prescribing Errors Based on Implementation Intentions: The Acceptability and Feasibility of My Prescribe. JMIR Hum. Factors.

[45] Morozov S., Guseva E., Ledikhova N., Vladzymyrskyy A., Safronov D. (2018). Telemedicine-based system for quality management and peer review in radiology. Insights Imaging.

[46] Orchard J., Li J., Freedman B., Webster R., Salkeld G., Hespe C., Gallagher R., Patel A., Kamel B., Neubeck L. (2020). Atrial Fibrillation Screen, Management, and Guideline-Recommended Therapy in the Rural Primary Care Setting: A Cross-Sectional Study and Cost-Effectiveness Analysis of eHealth Tools to Support All Stages of Screening. J. Am. Heart Assoc..

[47] Jury S.C., Kornberg A.J. (2016). Integrating telehealth in to ‘business as usual’: Is it really possible?. J. Telemed. Telecare.

[48] Glasgow R.E., Vogt T.M., Boles S.M. (1999). Evaluating the public health impact of health promotion interventions: The RE-AIM framework. Am. J. Public Health.

[49] Kidholm K., Jensen L.K., Kjølhede T., Nielsen E., Horup M.B. (2016). Validity of the Model for Assessment of Telemedicine: A Delphi study. J. Telemed. Telecare.

[50] Kidholm K., Clemensen J., Caffery L., Smith A. (2017). The Model for Assessment of Telemedicine (MAST): A scoping review of empirical studies. J. Telemed. Telecare.

[51] Kidholm K., Ekeland A.G., Jensen L.K., Rasmussen J., Pedersen C.D., Bowes A., Flottorp S., Bech M. (2012). A Model for Assessment of Telemedicine Applications: Mast. Int. J. Technol. Assess. Health Care.

[52] Kapoor L., Basnet R., Chand R.D., Singh S., Mishra S.K. An Audit of Problems in Implementation of Telemedicine Programme. Proceedings of the 2007 9th International Conference on e-Health Networking, Application and Services.

[53] Ndoro S. (2014). Effective multidisciplinary working: The key to high-quality care. Br. J. Nurs..

[54] Keers R.N., Williams S.D., Cooke J., Ashcroft D.M. (2013). Causes of Medication Administration Errors in Hospitals: A Systematic Review of Quantitative and Qualitative Evidence. Drug Saf..

[55] Chan J.C., Gupta A.K., Stewart S., Babidge W., McCulloch G., Worthington M.G., Maddern G.J. (2019). “Nobody told me”: Communication Issues Affecting Australian Cardiothoracic Surgery Patients. Ann. Thorac. Surg..

[56] Moore K. (2014). Improving communication between emergency department staff. Emerg. Nurse.

[57] Whitten P.S., Allen A. (1995). Analysis of Telemedicine from an Organizational Perspective. Telemed. J..

[58] Hargie O.D.W., Tourish D. (1993). Assessing the Effectiveness of Communication in Organisations: The Communication Audit Approach. Health Serv. Manag. Res..

[59] Goldhaber G., Rogers D. (1979). Auditing Organisational Communication Systems.

[60] Downs C. (1988). Communication Audits.

